# Estrogen receptor alpha deficiency protects against development of cognitive impairment in murine lupus

**DOI:** 10.1186/s12974-014-0171-x

**Published:** 2014-12-16

**Authors:** Melissa A Cunningham, Jena R Wirth, Linnea R Freeman, Heather A Boger, Ann-Charlotte Granholm, Gary S Gilkeson

**Affiliations:** Division of Rheumatology and Immunology, Department of Neurosciences, and Ralph H Johnson Veterans Affairs Hospital, Medical University of South Carolina, 96 Jonathan Lucas Street, Suite 814, MSC637, Charleston, SC 29425 USA

**Keywords:** Estrogen receptor alpha (ERα), Neuropsychiatric lupus (NP-SLE), Microglia

## Abstract

**Background:**

One of the more profound features of systemic lupus erythematosus (SLE) is that females have a 9:1 prevalence of this disease over males. Up to 80% of SLE patients have cognitive defects or affective disorders. The mechanism of CNS injury responsible for cognitive impairment is unknown. We previously showed that ERα deficiency significantly reduced renal disease and increased survival in lupus-prone mice. We hypothesized that ERα deficiency would be similarly protective in the brain, and that ERα may play a role in modulating blood-brain barrier (BBB) integrity and/or neuroinflammation in lupus-prone mice.

**Methods:**

MRL/lpr ERα+/+ and ERαKO mice (n = 46) were ovariectomized, received 17β-estradiol pellets, and underwent radial arm water maze (WRAM) and novel object recognition (NOR) testing starting at eight weeks of age. Mice were sacrificed and brains were hemisected and processed for either immunohistochemistry, or hippocampus and parietal cortex dissection for Western blotting.

**Results:**

MRL/lpr ERαKO mice (n = 21) performed significantly better in WRAM testing than wild-type MRL/lpr mice (n = 25). There was a significant reduction in reference memory errors (*P* <0.007), working memory errors (*P* <0.05), and start arm errors (*P* <0.02) in ERαKO mice. There were significant differences in NOR testing, particularly total exploration time, with ERα deficiency normalizing behavior. No significant differences were seen in markers of tight junction, astrogliosis, or microgliosis in the hippocampus or cortex by Western blot, however, there was a significant reduction in numbers of Iba1+ activated microglia in the hippocampus of ERαKO mice, as evidenced by immunohistochemietry (IHC).

**Conclusion:**

ERα deficiency provides significant protection against cognitive deficits in MRL/lpr mice as early as eight weeks of age. Additionally, the significant reduction in Iba1+ activated microglia in the MRL/lpr ERαKO mice was consistent with reduced inflammation, and may represent a biological mechanism for the cognitive improvement observed.

**Electronic supplementary material:**

The online version of this article (doi:10.1186/s12974-014-0171-x) contains supplementary material, which is available to authorized users.

## Background

Systemic lupus erythematosus (SLE) is the prototypic autoimmune disease characterized by production of autoantibodies and immune complex-mediated end-organ damage. Nine out of ten patients diagnosed with lupus are female, thus biologic sex is important in disease development [1]. The cause of the sex difference in SLE is likely multifactorial, including the sex chromosomes, sex hormones, and their receptors. A high incidence of neuropsychiatric involvement is being increasingly recognized. Symptoms of neuropsychiatric SLE (NP-SLE) are variable [2], however, certain symptoms are more common than others, with up to 80% demonstrating cognitive defects and/or affective disorders [3,4]. Neuroimaging of these patients, even those without clinical symptoms, reveals structural changes such as non-focal atrophy. On the other hand, cognitive behavioral deficits in lupus can be observed in the absence of gross central nervous system (CNS) pathology, and do not correlate well with serologic or disease activity measures [3,5]. Additionally, up to 40% of NP-SLE symptoms develop before or at the time of SLE diagnosis, and approximately 60% manifest within the first year [6,7]. Taken together, these data suggest a primary CNS-specific mechanism that is largely independent of disease flare or cumulative damage. The mechanism(s) of CNS injury responsible for the cognitive and psychological impairments in SLE are currently unclear.

Autoantibodies cross-reacting with N-Methyl-D-aspartate (NMDA) receptors in the brain mediate excitotoxic cell death, causing impairments in cognition and other behavioral changes in lupus-prone mice [8-10]. Similar disease manifestations are present in humans who have elevated cerebrospinal fluid (CSF) titers of these autoantibodies [11-13]. Biochemical studies in the affected mice revealed neuronal cell loss by apoptosis in targeted areas such as the hippocampus and lateral amygdala [8,14,15]. A breach in the blood-brain barrier (BBB) is required for these effects. Thus, BBB breakdown, pathogenic autoantibodies, and subsequent neuronal damage in key areas may be involved in the development of NP-SLE.

There is robust evidence that hormonal changes alter BBB integrity, which may predispose to different outcomes following pathological insult. Estrogen acts primarily via its receptors, ERα and ERβ, nuclear transcription factors involved in the regulation of many complex physiological processes. Thus, estrogen receptor modulators are being considered for a wide variety of conditions in humans [16]. Numerous studies have shown protective effects of estrogen in acute neurologic insult models (such as stroke, traumatic brain injury, and experimental autoimmune encephalomyelitis) [17-20]. In SLE and several other chronic inflammatory diseases, however, data suggest the opposite; estrogen has pro-inflammatory effects, such as enhancing humoral immunity, while progesterone and androgens are immunosuppressive [21-25]. Elevated estrogen levels may lead to prolonged or dysfunctional inflammatory responses. Our lab previously derived lupus-prone ERα-deficient (ERαKO) mice, and found that female MRL/lpr ERαKO and NZM2410 ERαKO mice developed significantly less proteinuria and pathologic renal disease, and had significantly prolonged survival [21]. We subsequently showed that innate immune cells (dendritic cells) from ERαKO mice have a blunted inflammatory response (for example, reduced levels of IL-6, MCP-1, and IL-1β to Toll-like receptor (TLR) ligands, partially explaining the protected phenotype [26].

Microglia are the innate immune cells of the CNS and respond to both external and internal stimuli with a cascade of reactive cytokines that can act in either a pro- or anti-inflammatory fashion [27]. Microglia originate from bone marrow-derived monocytes and migrate to the CNS, accounting for approximately 5 to 10% of CNS cells. Under normal conditions, microglia are actively surveying their environment, and after pathologic insult they become activated, undergoing dramatic changes in morphology and function, with concomitant increases in expression of CD11b and Iba1. Both ERα and ERβ are expressed in microglia, however ERα, but not ERβ, is required for activation, consistent with what is observed in peripheral innate immune cells [28,29].

Since female lupus-prone ERαKO mice are protected from renal disease, we investigated the effects of ERα deficiency on CNS disease. Given the known cognitive dysfunction in both human and murine lupus, we evaluated cognitive endpoints, specifically spatial and visual memory, following early ovariectomy and estradiol repletion to normalize estrogen levels in female lupus-prone wild-type (WT) and ERαKO animals. We subsequently examined the brains of these animals, with specific attention to the microglia, given the importance of ERα in innate immune cells. In this study, we show for the first time that the cognitive impairment seen in lupus-prone MRL/lpr mice is significantly improved in ERα-deficient littermates. Importantly, ERα-deficient mice have significantly fewer activated microglia in the CA1 and dentate regions of the hippocampus, despite no significant differences in autoantibody levels or systemic hormone levels, suggesting that ERα plays a critical role in the activation and/or development of immune cells involved in lupus CNS disease.

## Methods

### Mice

Mice were maintained at the Ralph H Johnson VA Medical Center Animal Facility (Charleston, South Carolina, United States). Animal protocols followed the principles outlined in the Guide for the Care and Use of Laboratory Animals, and were approved by MUSC’s IACUC (Institutional Animal Care and Use Committee), protocol #559. ERαKO mice of the C57BL/6 strain (kind gift of Dr Ken Korach) were backcrossed to MRL/lpr mice (Jackson Laboratory, Bar Harbor, Maine, United States) as previously described [21]. All experimental mice (n = 46) were female, either ERα+/+ or ERαKO, using littermates when possible. Mice were ovariectomized (OVX) using isoflurane (Patterson Veterinary, Devens, Massachusetts, United States) and held for two weeks to recover and eliminate endogenous ovarian steroids. At six weeks of age, a 0.25 mg 90-day time-release 17β-estradiol pellet (Innovative Research, Sarasota, Florida, United States, catalog number NE-121) was implanted sub-dermally in the dorsal neck. A 0.25 mg pellet results in serum levels averaging between 70 and 300 pg/ml.

### Radial arm maze apparatus and testing procedure

The radial arm water maze (WRAM) task is used to measure spatial (working and reference) memory [30-32]. For details of WRAM testing, please see Additional file 1. Briefly, animals were introduced to WRAM testing at eight weeks of age (two weeks after E2 pellet implantation). The eight-arm water maze was constructed of galvanized steel and filled with room-temperature water. See Hyde *et al*. for detailed information on procedure and a comparative analysis of three mouse strains (BXSB, NZB, and C57BL/6) [33]. The maze was located in a room with salient extra-maze cues on three walls, and the same experimenter in a yellow gown in the fourth position (start arm). For every trial, a subject was allowed 120 seconds to swim through the maze and locate a platform. Each subject received one session (four trials) per day for 12 days. A swim test, including velocity testing and sight testing, was conducted on day zero to ensure that animals were physically capable of performing in the maze. Data are presented as the average errors per session or per learning phase, similar to the methods described in Bimonte and Denenberg, and Alamed *et al*. [32,34].

### Novel object recognition

Recognition memory tasks utilize animal tendency to spend more time exploring novel objects compared to familiar objects. This task is known to involve both frontal cortex and hippocampal function [35]. For full details of novel object recognition (NOR) testing procedure, see Additional file 1. Briefly, on days one and two, the animal was habituated to the learning environment to reduce anxiety. On day three the animal was exposed to a three-minute familiarization session (identical objects present, A/A). The animal then performed a three-minute testing session after a 90-minute delay (A/B). On day four, the animal was tested with a second new object (A/C). The time to first contact, total amount of time spent with each object, as well as number of contacts were recorded and scored. The D^2^ index was calculated for an A/B or A/C session by examining the difference in time spent exploring the novel and familiar objects divided by the total exploration time for both objects:

$$ {\mathrm{D}}^2 = \left({\mathrm{T}}_{\mathrm{n}}\hbox{-}\ {\mathrm{T}}_{\mathrm{f}}\right)/\mathrm{Total}\ \mathrm{time}. $$

### Serum anti-dsDNA and serum estradiol

Serum was collected from WT and ERαKO female MRL/lpr mice at sacrifice. Serum anti-dsDNA was measured by ELISA assays as previously described [36]. Estradiol levels were assessed via ELISA (Calbiotech, San Diego, California, United States) [37]. The OVX + E2 mice averaged 379 pg/ml circulating estradiol. The sensitivity of the assay was 3 pg/ml; precision: 3.1% (intra-assay), 9.9% (inter-assay) [38].

### Western blotting

At the time of sacrifice, brains were quickly removed and hemisected. One hemisphere was post-fixed for immunohistochemistry. The other hemisphere was dissected to harvest hippocampus, pre-frontal cortex, nucleus accumbens, and a piece of parietal cortex, all of which were snap frozen and stored at -80°C. Tissue was sonicated in a modified RIPA (150 mM NaCL, 50 mM Tris-HCl, pH 7.4, and 0.1% sodium dodecyl sulfate) buffer with protease (Thermo Scientific, Rockford, Illinois, United States) and phosphatase inhibitors (Sigma, Saint Louis, Missouri, USA). Lysates were analyzed by 10% SDS-PAGE and transferred to a polyvinylidene fluoride membrane (Life Technologies - Invitrogen, Grand Island, New York, United States). After blocking, the membrane was incubated with rabbit anti–tight junction protein Zo-1 (Invitrogen, number: 61-7300), anti-occludin (Invitrogen, number: 40-4700), anti-Iba1 (Wako, Richmond, Virginia, United States, number: 019-19741), anti-Microtubule Associated Protein 2 (MAP2) (Abcam, Cambridge, Massachusetts, United States, number: 32454), or mouse anti- Glial fibrillary acidic protein (GFAP) (Cell Signaling, Danvers, Massachusetts, United States, number: 3670).

### Immunofluorescence

For immunohistochemical analysis, one hemisphere was post-fixed in 4% paraformaldehyde for 48 hours, and then transferred to 30% sucrose (Thermo Fisher Scientific, Waltham, Massachusetts, United States, number: S5500) in 0.1 M phosphate buffered saline (PBS) and 0.1% sodium azide (Thermo Fisher Scientific, number: S2271-500) before sectioning. The hemisphere was sectioned on a cryostat (Microm, Zeiss, Dublin, California, United States) at 40 μm and processed for immunohistochemical analyses as described previously [39]. Briefly, series of every sixth section were blocked in 10% normal goat serum (NGS, Invitrogen, number: 10000C) in Tris Buffered Saline (Thermo Fisher Scientific, number: BP152-5)-Triton X-100 (Thermo Fisher Scientific, number: BP151-100) and incubated with anti-Iba1 (Abcam, number: 153696), Alexa-fluor 488 conjugated rabbit polyclonal anti-Neu1 (Millipore, Billerica, Massachusetts, United States, number: ABN78A4), or Alexa-fluor 488 conjugated mouse monoclonal antibody against MAP2 (Millipore, number: MAB3418X) overnight at room temperature. The sections were then incubated for one hour with secondary antibodies directed against the appropriate species, conjugated with tetramethylrhodamine (TRITC, 1:200) (Jackson Immunoresearch, West Grove, Pennsylvania, United States). The tissue was mounted onto slides, and cover-slipped with an anti-fade solution (Fisher Scientific, Fair Lawn, New Jersey, United States). All images we captured using a Nikon Eclipse E-600 microscope equipped with a Qcam digital camera (Nikon Instruments Inc., Melville, New York, United States).

### Iba1 cell counts

The density of Iba1 immunoreactive microglial cells in the hippocampus was investigated because of this brain region’s proven role in both of the behavioral tasks employed herein. Our group has previously demonstrated significant alterations in microglial activation in the CA1 and dentate regions of the hippocampus in other chronic inflammatory conditions, such as following long-term exposure to a high-fat diet [40]. A series of every sixth section throughout the rostrocaudal extent of the hippocampus was evaluated. Images were acquired on a Nikon microscope, and settings remained identical across all data acquisition. Using the NIS-Elements Advanced Research software system from Nikon (Nikon Instruments Inc.), the region of interest (ROI) was outlined and the number of Iba1 immunoreactive cells was counted by an investigator blinded to the group identity. The number of Iba1 immunoreactive cells was expressed as the average number of cells observed within one field of vision using a 10× objective, averaged across three randomly selected visual fields within each tissue section. The results were averaged per animal and data analyzed using an unpaired Student’s *t*-test (Graphpad, La Jolla, California, United States) between the two groups.

### Statistical analysis

For WRAM studies, repeated measures ANOVA was utilized for learning curves, and a Mann-Whitney nonparametric *t*-test was utilized to test for significance between the two mouse groups. For NOR studies, one-way ANOVAs were performed followed by *post-hoc* analysis with Tukey’s multiple comparison testing. Iba1 immunoreactive cell counts were analyzed using an unpaired Student’s *t*-test. Standard error of the means (SEM) was reported where applicable. *P* values ≤0.05 were considered significant. All statistical analyses were performed using GraphPad Prism Version 5.0 Software.

## Results

### Hormone and autoantibody levels

**Figure 1:**
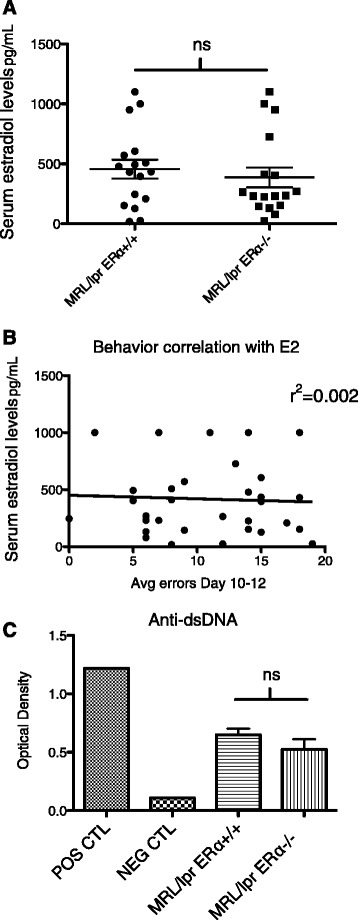
**Hormone and autoantibody levels. (A)** There was no significant difference in average 17β-estradiol level between the MRL/lpr WT and MRL/lpr ERαKO groups of mice. **(B)** There was also no correlation between individual serum estradiol levels and behavior (performance in the WRAM). **(C)** There was no significant difference between MRL/lpr WT and ERαKO mice with regard to anti-dsDNA levels at 10 weeks of age. POS = single sample from a sick/aged MRL/lpr WT; NEG = single sample from a B6 mouse.

MRL/lpr ERα WT and ERαKO female mice were OVX at three to four weeks of age and an estrogen pellet was placed at six weeks of age. Estradiol pellets (extended release) were designed to approximate physiologic levels of estradiol (approximately 100 ng/ml/day). Given that estrogen improves performance by rodents in the WRAM [41], we confirmed that there were no significant differences in serum estradiol levels between the MRL/lpr and MRL/lpr ERαKO mice (Figure 1A). There was also no correlation between estradiol levels in individual mice and performance in the WRAM, as measured by late (days 10 to 12) reference memory errors (Figure 1B).

Overall lupus disease burden of MRL/lpr and MRL/lpr ERαKO mice was previously characterized by our laboratory [21]. Despite significant differences in renal disease, non-manipulated ERαKO female animals did not have diminished serum autoantibody levels. In this study, using OVX MRL/lpr mice (WT and ERαKO), anti-dsDNA levels were again not significantly different (Figure 1C). These results suggest that variance in autoantibody and hormone levels by themselves do not explain the behavior differences noted in these two groups of mice.

### Radial arm water maze testing

The WRAM task utilized here tests spatial reference and working memory [42], while the NOR task has been associated with both hippocampal and frontal cortex function [35]. During the habituation trial on day one, swim tests were performed by all animals (48 cm to unconcealed center platform with large flag). Animals took approximately two seconds to reach the center platform after release and no impairments in swimming speed or ability to utilize visual cues were observed in either MRL/lpr or MRL/lpr ERαKO mice (1.76 ± 0.35 versus 2.19 ± 0.94 seconds, respectively).

**Figure 2:**
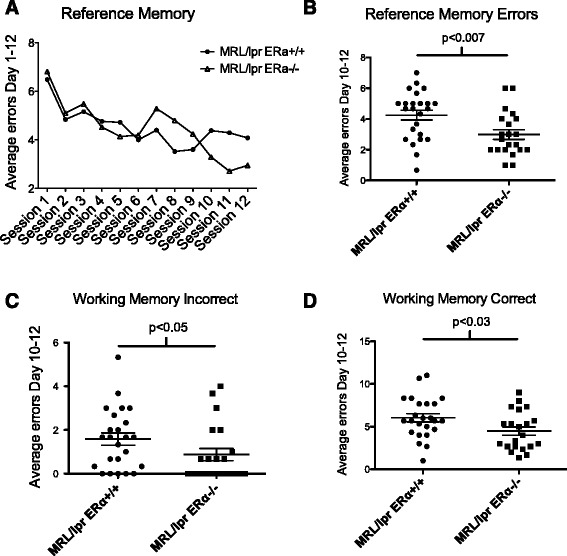
**MRL/lpr ERαKO mice perform better than MRL/lpr WT in WRAM testing. (A)** Reference memory learning curve. The one-within (session) repeated measures revealed a significant effect of sessions for days 1 to 12. **(B)** In the late or asymptotic phase (days 10 to 12), MRL/lpr ERαKO mice outperformed MRL/lpr mice, with significantly fewer reference memory errors made. **(C, D)** Average errors made in the late or asymptotic phase (days 10 to 12) revealed that MRL/lpr ERαKO mice made significantly fewer errors than MRL/lpr mice in both working memory incorrect and working memory correct errors.

Four orthogonal measures of reference and working memory were obtained for each daily session: working memory correct errors, reference memory errors, working memory incorrect errors, and start arm errors. Reference memory errors were the number of first entries into an arm that had never contained a platform. Additional file 2: Table S1 lists the N (number of animals), means +/- SE (standard error) for the number of errors made. The one-within (session) repeated measures ANOVA (*F*s_1,11_ = 4.09; *P* <0.01) revealed a main effect of sessions while a *t*-test (genotype) for days 1 to 12 did not reveal a significant effect of genotype, most likely due to the increased errors seen in MRL/lpr ERαKO mice mid-training. Figure 2A shows the learning curve. As expected, there was no significant difference between the two groups early in training (the acquisition phase), however, by the end of training (the asymptotic phase), MRL/lpr ERαKO mice clearly outperformed MRL/lpr mice, with significantly fewer errors made in the late or asymptotic phase (days 10 to 12; *P* <0.007) (Figure 2B).

Working memory incorrect (WMI) errors were the number of repeat entries into an arm that never contained a platform. Additional file 2: Table S2 lists the the N, means +/- SE for the number of errors made. There was no significant difference between the two groups early in training, but again by the end of training, MRL/lpr ERαKO mice improved their average error rate, with significantly fewer errors made (days 10 to 12) versus MRL/lpr mice (*P* <0.05) (Figure 2C). Similar performance between the two groups in the acquisition phase suggests that there was no significant difference between the groups in terms of motivation to learn or perform the task, thus providing some information regarding mood and/or motivation. Working memory correct errors were scored as the number of first and repeat entries into an arm of the maze where a platform had been removed during the session. Additional file 2: Table S3 lists the N, means +/- SE for the number of errors made by MRL/lpr versus MRL/lpr ERαKO mice. There was a significant difference between the two groups in average errors made in the late/asymptotic phase of training (days 10 to 12). MRL/lpr ERαKO made significantly fewer errors than MRL/lpr mice (*P* <0.03) (Figure 2D), suggesting that MRL/lpr ERαKO mice showed enhanced learning for the task. It should be noted, however, that the learning curves for both groups of mice suggest that neither group reduced the error rate to the extent expected for a normal mouse [32,43].

**Figure 3:**
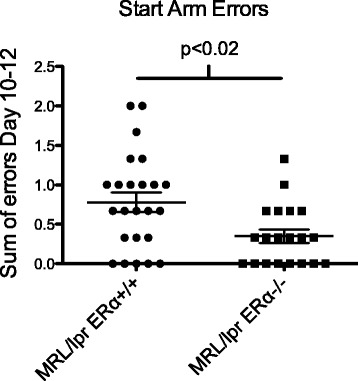
**WRAM start arm errors.** MRL/lpr mice exhibit abnormal behavior. ERαKO mice made significantly fewer errors versus MRL/lpr mice.

Lastly, start arm errors were recorded as any entry in arm five, which is the release arm. Since the examiner sits at the end of this arm, there is a strong visual cue. Animals must learn not to return to their release point, as it never holds a platform. Again, MRL/lpr ERαKO mice made significantly fewer errors (days 10 to 12) versus MRL/lpr mice (*P* <0.02) (Figure 3).

### Novel object recognition testing

The NOR task is utilized to detect improvement (or disturbance) of non-spatial memory [44]. It requires a capacity for both identification and judgment of the prior occurrence of what has been identified. This visual recognition memory is evolutionarily conserved among species, and requires the hippocampus [45,46].

**Figure 4:**
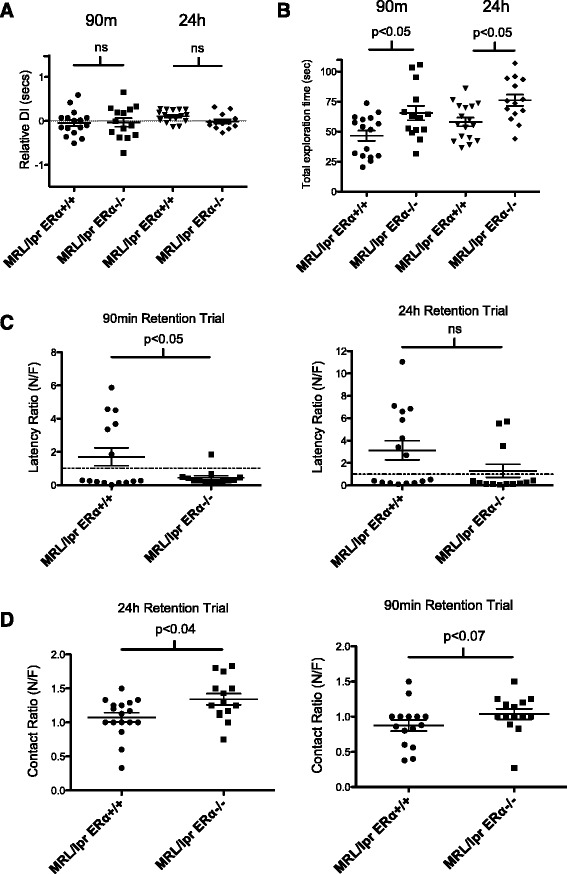
**MRL/lpr ERαKO mice perform better than MRL/lpr WT in novel object recognition testing. (A)** Relative discrimination (N-F/T) index. There was no significant difference between the discrimination ability of the MRL/lpr versus MRL/lpr ERαKO at 90 minutes or 24 hours. Neither group could effectively discriminate between the novel and familiar objects by this measure. A relative discrimination index of 0% (dotted line) indicates chance performance. **(B)** There was a significant difference in total exploration times between MRL/lpr and MRL/lpr ERαKO mice in both 90-minute and 24-hour trials. *Post-hoc* analysis revealed the total exploration time in both T1 and T2 of MRL/lpr ERαKO mice was higher than the total exploration time of MRL/lpr mice. **(C)** The latency ratio (time to first contact) was significantly decreased in the MRL/lpr ERαKO versus MRL/lpr mice at 90 minutes. There was also a trend towards improved performance in MRL/lpr ERαKO at 24 hours. **(D)** Contact ratio: MRL/lpr ERαKO mice made significantly more nose contacts with the novel object than the familiar object at 24 hours, and at 90 minutes there was a trend towards significance.

Analysis of performance by standard NOR formulas (recognition index (RI) and relative discrimination index) revealed that there was no significant effect of genotype on the overall discrimination performance during 90-minute and 24-hour retention trials (*F*(3, 55) = 1.125, ns). Figure 4A shows the relative discrimination (N-F/T) index. A relative discrimination index of 0% (dotted line) indicates chance performance. There was a non-significant trend towards increased discrimination ability of the MRL/lpr versus MRL/lpr ERαKO at 24 hours (*P* <0.07). Although there were individuals who could perform the task, neither group, as a whole, could effectively discriminate between the novel and familiar objects, suggesting abnormal behavior in both groups. This is in contrast to what was shown by other groups comparing MRL/lpr to MRL/+ mice [47,48] in which MRL/lpr mice did not exhibit abnormal behavior in this task.

An unexpected result was the significant difference in total exploration times between MRL/lpr and ERαKO mice in both the 90 minute and 24 hour trials (strain: *F*(3, 57) =7.135; *P* <0.0004). *Post-hoc* analysis revealed that the total exploration time in both T1 and T2 of MRL/lpr ERαKO mice was significantly higher than that of MRL/lpr mice (*P* <0.05) (Figure 4B).

When measuring latency (time to first contact) and contact ratio (number of contacts with novel/familiar), there was improved recognition of novel versus familiar object in the MRL/lpr ERαKO mice. At 90 minutes, the latency ratio (time to novel/time to familiar) was significantly decreased in the MRL/lpr ERαKO mice (*P* <0.05) (Figure 4C). There was a trend towards improved behavior of MRL/lpr ERαKO at 24 hours. The percent of MRL/lpr ERαKO mice with a latency ratio less than one (novel preference) was 92% (12 out of 13) versus only 60% (9 out of 15) of MRL/lpr mice. Similarly, at 24 hours, 77% (10 out of 13) of MRL/lpr ERαKO had a latency ratio less than one versus only 50% (8 out of 16) of MRL/lpr mice. With regard to nose contacts, MRL/lpr ERαKO mice made significantly more contacts with the novel object than the familiar object at 24 hours (*P* = 0.04), and at 90 minutes there was a trend towards significance (*P* = 0.07) (Figure 4D). In total, 43% (6 out of 14) of ERαKO mice made more contacts with the novel versus familiar object, whereas only 13% (2 out of 15) of the WT MRL/lpr did so after a 24-hour delay. These results suggest that there was improved recognition of novel versus familiar objects by MRL/lpr ERαKO mice. While the behavior is not normal in either group, ERα deficiency improved their performance in NOR testing.

### Global hippocampal expression of tight junction proteins Zo-1 and occludin

Autoantibodies cross-reacting with NMDA receptors in the brain can mediate excitotoxic neuronal cell death, which causes impairments in cognition and other behavioral changes in lupus-prone mice [8-10]. A breach in the BBB is required for these effects. Estrogen, in multiple studies, protects the BBB in both normal aging and in certain disease states, and this effect is dependent on ERα or β [20,49,50].

To test whether BBB permeability may play a role in the cognitive differences seen in MRL/lpr versus MRL/lpr ERαKO animals, we measured several tight junction proteins that are surrogate markers of BBB integrity using Western blot analysis. We prepared whole cell lysates of the hippocampus from MRL/lpr and ERαKO mice (n = 9 WT, 9 ERαKO) and did Western blot analysis for Zo-1 or occludin. We did not see any global change in Zo-1 (Additional file 2: Figure S1A) or occludin (data not shown) at 10 weeks of age in the hippocampus or cortex of MRL/lpr versus ERαKO mice, although there was a trend towards reduced Zo-1 in MRL/lpr ERαKO mice. These findings do not exclude potential morphological alterations at the microstructural level of BBB integrity in any of the groups as a result of the manipulation.

### Global hippocampal expression of GFAP, Iba1, and MAP2

Neurologic disorders often lead to activation of resident microglia and invasion of blood-borne macrophages, which are accompanied by an increase in number and change in phenotype of astrocytes, a phenomenon generally termed reactive astrocytosis. We previously demonstrated that ERα plays a role in modulating inflammation in lupus-prone mice [26]. We hypothesized that a potential role for ERα in CNS disease is by modulating inflammatory mediators that lead to astrocytosis, microgliosis, and/or neuronal/dendritic loss. To test this hypothesis we prepared whole cell lysates of the hippocampus from MRL/lpr and ERαKO mice (n = 18) and did Western blot analysis for GFAP (an integral protein in astrocytes), Iba1 (marker of microgliosis), and MAP2 (marker of neuronal growth, plasticity, and degeneration). We did not see global changes in these markers at 10 weeks of age in the hippocampus (Additional file 2: Figure S2A,B,C) or cortex (data not shown). There was a trend towards decreased Iba1 in the cortex of MRL/lpr ERαKO mice, but it did not reach significance. Protein analysis of whole tissue homogenates may not always detect discrete differences observed in specific cell populations in the brain, making morphological assessment of both glial markers necessary.

### Central nervous system inflammatory changes

**Figure 5:**
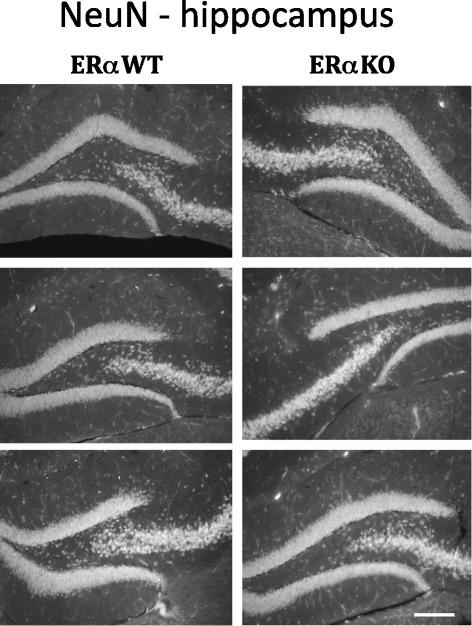
**Hippocampal NeuN staining - MRL/lpr and MRL/lpr ERαKO mice.** Selected images of NeuN immunostaining in the hippocampus of MRL/lpr and MRL/lpr ERαKO mice showing CA1 and dentate regions of interest (n = six to eight per group evaluated, three representative animals shown). There were no significant differences in hippocampal structure or volume noted. Scale bar in lower right corner represents 150 microns.

Given our previous data, we hypothesized that ERα deficiency would decrease inflammation in the brain, leading to protection against neuronal loss (correlating with improved behavior). Utilizing immunohistochemistry to assess hippocampal structure, we noted that several animals had hippocampal (CA1 thinning) or cortical lesions, but these lesions did not correlate with ERα status or behavior. Overall, there were no significant differences seen in hippocampal structure utilizing NeuN immunostaining (a marker for neuronal cell bodies, Figure 5) or MAP2 immunostaining (a marker for dendritic integrity, Additional file 2: Figure S3), although multiple MRL/lpr animals had reduced overall MAP-2 signal in the hippocampus, suggesting reduced numbers of dendrites and dendritic complexity in MRL/lpr versus MRL/lpr ERαKO mice.

**Figure 6:**
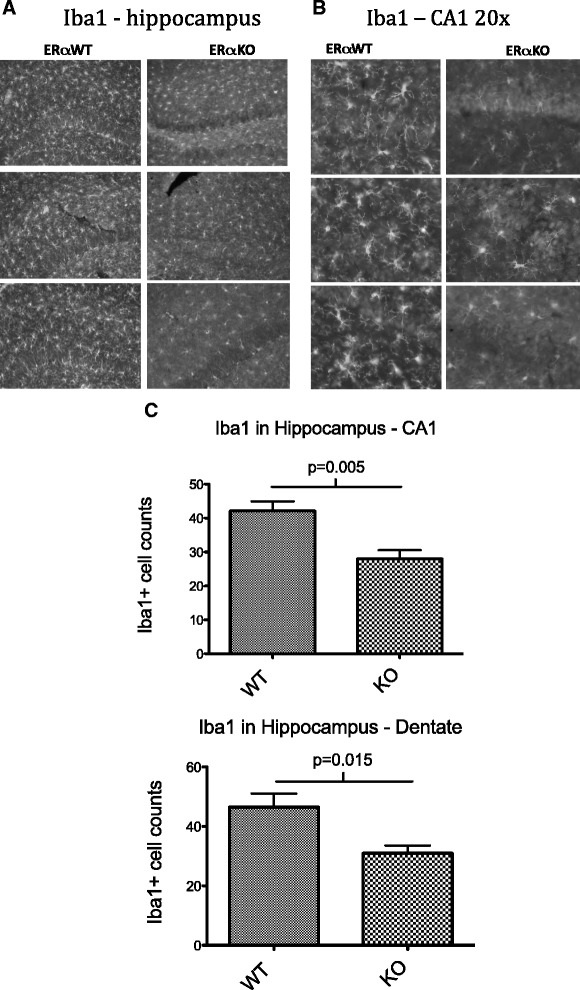
**Hippocampal Iba1 staining in MRL/lpr and MRL/lpr ERαKO mice. (A)** IHC images demonstrate diffuse glial activation and clustered microglia in the hippocampus of both MRL/lpr and MRL/lpr ERαKO mice. Scale bar in lower right corner represents 100 microns. **(B)** Higher magnification (20×) images demonstrating increased Iba1 immunopositive cells in MRL/lpr WT versus ERαKO mice. Scale bar in lower right corner represents 50 microns. **(C)** Counts of Iba1+ cells in CA1 and dentate regions of hippocampus showing a significant reduction in Iba1+ cells in MRL/lpr ERαKO mice (n = six to eight mice per group, average of three random fields each).

As described above, there were no global differences in microgliosis in the hippocampus or cortex seen by Western blot analysis. We subsequently examined localized changes by immunohistochemistry using the microglial marker Iba1. Again, gross hippocampal and cortical abnormalities were found in both groups. Almost all animals had some level of inflammation in both the hippocampus and the cortex, as evidenced by diffuse activated astrocytes or clustered microglia (Figure 6), however the level of microglial activation appeared to be significantly different between WT MRL/lpr and MRL/lpr ERαKO animals, based on the Iba1 immunohistochemistry. When semi-quantitative cell counts were undertaken in regards to Iba1 immunoreactivity, significant differences in both number and intensity of Iba1 staining within each microglial cell were observed. Hippocampal sections from MRL/lpr ERαKO had, on average, significantly fewer activated microglia compared with Iba1 immunoreactive microglia in the WT MRL/lpr group (*P* = 0.005 – CA1, *P* = 0.015 – dentate, Figure 6C). The Iba1 data indicate that a potential mechanism for the cognitive protection in MRL/lpr ERαKO mice may be reduced inflammation or microgliosis.

## Discussion

Of the one and a half million Americans afflicted with lupus, 90% are women, the vast majority of whom are between the ages of 15 and 45 years, when they are most hormonally active [51]. Approximately 60% of SLE patients have CNS or peripheral nervous system involvement, ranging from cognitive defects and mood disorders to severe presentations such as psychosis, seizure, stroke, and coma [52]. We hypothesized that ERα plays a role in NP-SLE by mediating local inflammation and/or modulating the BBB, and that ERα deficiency would protect against development of NP-SLE symptoms, based on the renal protection and survival benefit we previously reported in lupus ERαKO mice. Our current data contribute to the previous literature by demonstrating that a mouse model of SLE (MRL/lpr mice) was protected from cognitive impairment when paired with an ERαKO mouse model, and our findings strongly suggest that cognitive improvement observed in the MRL/lpr ERαKO mice may be due to reduced activation of microglia in the hippocampus. Our laboratory previously showed that ERα is a key mediator of SLE in female lupus-prone mice (both NZM2410 and MRL/lpr) [21]. Deficiency of ERα in these mice resulted in significantly reduced renal disease and increased survival. This protective effect was independent of autoantibody production and immune complex deposition. These results indicate that ERα may be important in local modulation of the inflammatory response. In the present study, we investigated the effects of ERα deficiency in the brains of these mice. We analyzed cognitive endpoints in OVX lupus-prone mice, with estradiol replacement to control for the aberrantly high levels of estradiol and testosterone normally found in ERαKO mice.

Similar to human lupus, MRL/lpr disease expression includes behavioral and neurologic dysfunction. The onset correlates with puberty and therefore raises the question of whether hormonal changes contribute to the disease process. Our findings herein are consistent with previous studies showing that MRL/lpr mice exhibit spatial memory deficits, as shown by weak performance in the Morris water maze and in linear maze acquisition [53,54]. In our current study, MRL/lpr mice were tested in a WRAM that does not rely on food reinforcement or food-seeking behavior (which could be confounded by differences in hormonal state or motivation). The WRAM also provides additional information over the Morris water maze regarding how the animals learn and recall as memory load increases, and therefore provides information regarding both working and reference spatial memory [55]. Consistent with previous maze studies in non-manipulated MRL/lpr mice, our OVX E2-replete MRL/lpr mice performed poorly in memory testing, with abnormal learning curves in the WRAM regardless of the type of memory error.

The most obvious deficits were in late training periods (days 10 to 12) when MRL/lpr performed nearly as poorly as they did at days one to three and days four to six, suggesting that the vast majority of animals had not been able to ‘learn the task’. The deficits observed in MRL/lpr mice in this study were quite severe compared with the milder deficits seen in prior maze studies. One explanation is that the WRAM is a more rigorous test of spatial memory and may be more sensitive to detect impairment. Nonetheless, MRL/lpr ERαKO exhibited superior learning ability by all four measures of memory type, suggesting that lack of a functional ERα protects against full development of cognitive impairment in this disease model.

While there is a long line of evidence to support estradiol’s role in improved memory and cognition, it is unlikely that ovariectomy alone explains the impaired performance in MRL/lpr, since estradiol levels were replete to physiologic levels. Importantly, estradiol levels did not correlate with behavior in either group, suggesting that estrogen alone could not rescue the phenotype. Thus, estradiol did not impact cognition via another (intact) receptor (such as ERβ). It is possible that cyclical estrogen exposure (mimicking physiologic levels) might result in different outcomes (versus the continuous E2 dosing experienced by these animals), but this would not be expected in the absence of ERα.

In addition to the WRAM, we utilized NOR testing to further study memory and cognition in these animals. NOR is attractive because it is a simple memory assay that relies mainly on the animal’s innate exploratory behavior, without training or outside reinforcement [56], and relies on functional integrity both of the hippocampus and the frontal cortex [35]. The preference for the novel object relies on the supposition that the familiar object is remembered. Neither MRL/lpr nor MRL/lpr ERαKO mice could (as a group) reliably distinguish between objects at a short (90 minute) or long (24 hour) time point by relative discrimination index. This is in contrast to a previous study that revealed no significant deficit in NOR testing in MRL/lpr mice, although mice were tested with a shorter delay (45 minutes) in that study [47]. Our assessment was also more stringent in that rearing was not counted, and snout distance had to be ≤1 cm (versus 3 cm).

There were, however, significant differences in total exploration time and object contacts between the groups, revealing a normalized propensity of MRL/lpr ERαKO mice to explore. Perhaps the best-studied neuropsychiatric abnormality in the MRL/lpr strain is the depressive-like behavior observed as early as five to six weeks of age. Using forced swim tests, multiple studies have shown ‘behavioral despair’ in these animals, as evidenced by voluntary immobility (floating), whereas normal animals swim vigorously to attempt escape [47,48,57,58]. Other symptoms of depressive behavior in these animals include anhedonia, apathy, and decreased activity. In rodents, the latter is often assessed as decreased exploration in a novel environment. In fact, MRL/lpr mice exhibit abnormal behavior in open field testing [53,58-60]. Thus, it is possible that the decreased exploration time, and lack of interest in the objects as a whole, is an indication of altered emotional reactivity, confounding the memory and cognitive aspect of the task. MRL/lpr ERαKO mice, in contrast, exhibited normal exploratory behavior in a novel environment. It is also possible that the significantly increased start arm errors committed by MRL/lpr mice in the WRAM were due to their tendency to return to the familiar, rather than explore the unknown. The behavioral performance in the earlier trials (trial one and two) within each day of the WRAM can also be used to detect motivation and/or mood disturbance. If mice perform poorly in early trials, this would suggest either inability or reduced motivation to swim and find the escape platform. Because the performance in the early trials of the WRAM did not appear to be different between the groups, this would suggest that motivation or mood were not affecting their ability to perform this task, while the NOR data instead suggest reduced exploratory behavior. Together, the WRAM and NOR testing suggest that MRL/lpr mice have both cognitive and emotional reactivity deficits, beginning at an early age, and that lack of a functional ERα is protective.

Studies in rodents and primates demonstrated that the hippocampus and perirhinal cortex are important for spatial and visual object recognition memory [61-64]. The CA1 zone of the hippocampus appears particularly vulnerable to behavior-related changes, and Sakic *et al*. found that CA1 pyramidal neuron dendrites atrophy in MRL/lpr mice [65]. This correlates with work done by DeGiorgio *et al*. that also showed specific degeneration of glutamate-receptor-positive neurons in the CA1 hippocampus after injection of the R4A autoantibody [8]. These previous findings motivated our decision to explore morphologic alterations in this particular brain region. In terms of global brain changes, MRL/lpr brain morphology studies documented enlarged ventricles and reduced parenchymal weights [65-67].

We examined the hippocampus and cortex from 20 MRL/lpr and MRL/lpr ERαKO mice and found that animals from both groups exhibited inflammatory changes in brain parenchyma as indicated by microgliosis, but that microgliosis was markedly attenuated in ERαKO brains. Western blot analysis did not reveal a significant difference in Iba1 between the two groups, which may be secondary to the insensitivity of this assay to pick up focal changes and subtle differences in expression patterns within a particular cell type in that brain region, the sum of which may have a significant impact on disease state. Overall, the effects of ERα receptor activation are likely multifactorial, but these data suggest that there is indeed an ERα-mediated inflammatory change in this model of chronic inflammation.

With regard to ER (and estrogen) beneficial or detrimental effects in the brain, the literature is extensive, but also full of inconsistencies, likely due to the pleiotropic nature of estrogen depending on disease state, cell type, dosing, timing, receptor-activating pathways, and so forth. Estradiol is highly lipophilic and can easily pass the BBB to modulate neuronal activity, particularly in the limbic system, which is rich in estrogen receptors. A role for estrogen receptors in neuroinflammation has been recognized. The selective estrogen receptor modulator (SERM) raloxifene, for example, can elicit robust neuroprotection against experimental autoimmune encephalomyelitis, partially via an inhibitory action on NF-κB and CCL20, a chemokine involved in neuroinflammation [68].

Our understanding of the pathophysiology of CNS lupus and the role of microglia in human disease is extremely limited. In a small series of seven patients with fatal neuropsychiatric lupus, widespread changes in the histologic features of the brain were present, including microinfarcts, microhemorrhages, thrombotic angiopathy, neuronal necrosis, reduced numbers of axons and neurons, and reactive microglia [69]. *In vitro*, microglia from primary cultures of human embryonic CNS cells, are the main producers of IL-6, IL-1β, and tumor necrosis factor alpha (TNFα) following stimulation with Toll-like receptor (TLR) 4 ligand or IL-1β [70]. In mice, brain atrophy, increased activation of microglia, and condensation of cytoplasm suggest a metabolic perturbation (for example, excitotoxic damage) that causes dysfunction and early death of central neurons during lupus-like disease [71]. Several investigators have demonstrated that ERα receptors are expressed by glial cells, and it has been shown that glial ERα expression increases with age [72]. This group also demonstrated that lowering ERα reversed aging-associated gliosis in the cortex, and partially restored E2-dependent neurite outgrowth, suggesting that ERα overexpression may be detrimental for neuroplasticity, and give rise to microglial activation. Although estrogen can inactivate microglia and inhibit the recruitment of T cells and macrophages into the CNS, there is controversy regarding which of the two estrogen receptors (ERs), ERα or ERβ, mediates the beneficial effects in microglia. Our studies provided herein, suggest that the two estrogen receptors may have opposing effects on chronic neuroinflammation, since previous studies have indicated that ERβ agonists can suppress microglial activation in the brain [73], while our current findings indicate that a genetic knockout of the ERα receptor (leaving ERβ unopposed) has a similar effect, and therefore acts as a beneficial down-regulator of microglial response in our mice. Further studies will reveal whether ERαKO resulted in reduced activation of microglia via the classical and/or alternative activation pathways (which have diametrically opposed effects on brain physiology and behavior) [74]. Early development and migration of microglia to the CNS are also likely impacted by ERα deficiency since these cells originate from bone marrow myeloid progenitors that normally require ERα for expansion and differentiation [75,76].

## Conclusions

Major efforts in the field are devoted to understanding estrogen influences on learning, memory, and mood, as well as the mechanisms that mediate these brain activities in both normal and disease states. The present study demonstrates that lack of ERα improves cognitive and emotional deficits of NP-SLE in female lupus-prone mice. The mechanism of this affect is not fully elucidated. The current study suggests that deletion of a functional ERα suppresses the pro-inflammatory actions of microglia and may contribute to reduction of sustained activation of microglia in this autoimmune disease. In humans, NP-SLE symptoms can be an early and devastating aspect of the disease. Further defining the effects of ERα in NP-SLE will provide new insight into mechanisms of disease and provide novel approaches to therapy.

## Additional files

Additional file 1 **Supplementary Materials and methods.**

Additional file 2: Table S1-S3. (1) Summary of reference memory errors: 86% of MRL/lpr ERKO mice improved (vs. 42% of WT). (2) Summary of working memory incorrect errors: 74% of MRL/lpr ERKO mice improved (vs. 48% of WT). (3) Summary of working memory correct errors: 62% of MRL/lpr ERKO mice improved (vs. 46% of WT). **Figure S1.** Western blot analysis of whole cell lysates from hippocampus and cortex of MRL/lpr and MRL/lpr ERαKO mice for Zo-1. There were no significant global changes in Zo-1 at 10 weeks of age in the hippocampus or cortex of MRL/lpr vs. MRL/lpr ERαKO mice although there was a trend towards reduced Zo-1 in MRL/lpr ERαKO mice. **Figure S2.** Western blot analysis of whole cell lysates from hippocampus of MRL/lpr and MRL/lpr ERαKO mice (n=9 WT, 9 KO) for **(A)** GFAP (marker of astrocytosis), **(B)** Iba1 (marker of microgliosis), and **(C)** MAP2 (marker of neuronal growth, plasticity, degeneration). There were no significant global changes in these markers at 10 weeks of age in the hippocampus of MRL/lpr vs. MRL/lpr ERαKO mice. **Figure S3.** Hippocampal MAP2 staining in MRL/lpr and MRL/lpr ERαKO mice. IHC images demonstrate multiple MRL/lpr animals had reduced overall MAP-2 signal in the hippocampus, suggesting reduced numbers of dendrites and dendritic complexity in MRL/lpr versus MRL/lpr ERαKO mice. Scale bar in lower right corner represents 50 microns. **Figure S4.** Hippocampal Iba1 staining in MRL/lpr and MRL/lpr ERαKO mice. IHC images demonstrate diffuse glial activation and clustered microglia in the dentate of both MRL/lpr and MRL/lpr ERαKO mice. These 20× images demonstrate increased Iba1 immunopositive cells in MRL/lpr WT versus ERαKO mice. Scale bar in lower right corner represents 50 microns.

## Abbreviations

BBB: blood-brain barrier
CA1: Cornu Ammonis area 1
CCL20: chemokine ligand 20
CNS: central nervous system
CSF: cerebrospinal fluid
dsDNA: double stranded deoxyribonucleic acid
ERα: estrogen receptor alpha
ERβ: estrogen receptor beta
GFAP: glial fibrillary acidic protein
Iba1: induction of brown adipose 1
IL1β: interleukin 1 beta
IL6: interleukin 6
MAP2: microtubule associated protein 2
MCP1: monocyte chemoattractant 1
MRL/lpr: Murphy Roths Large/lymphoproliferative
NFkB: nuclear factor kappa-light-chain-enhancer of activated B cells
NMDA receptors: N-Methyl-D-aspartate receptors
NOR: novel object recognition
NP-SLE: neuropsychiatric lupus
OVX: ovariectomy
SERM: selective estrogen receptor modulator
SLE: systemic lupus erythematosus
TLR: Toll-like receptor
TNFα: tumor necrosis factor alpha
WRAM: radial arm water maze
