# Supplementary material for: Estrogen receptor alpha deficiency protects against development of cognitive impairment in murine lupus
Source: J Neuroinflammation. 2014 Dec 16;11:171. doi: 10.1186/s12974-014-0171-x (PMC4272530; doi:10.1186/s12974-014-0171-x)
Supplement: Supplementary file 1 — Supplementary Materials and methods. [file 12974_2014_171_MOESM1_ESM.docx]

**Radial arm maze apparatus and testing procedure**

The radial arm water maze (RAWM) task can be used to measure both spatial working and reference memory [[1-3](#_ENREF_1)]. Animals were introduced to RAWM testing at 8 weeks of age (4 weeks after OVX and 2 weeks after E2 pellet implantation). The 8 arm water maze was constructed of galvanized steel and filled with room-temperature water. See Hyde *et al.* for detailed information on procedure and a comparative analysis of three mouse strains (BXSB, NZB and C57BL/6) [[4](#_ENREF_4)]. The maze was located in a room with salient extra-maze cues on 3 walls, and the same experimenter in a yellow gown in the 4^th^ position (start arm). Hidden escape platforms with wire mesh tops (one cm below the water surface) were placed at the ends of four of the eight arms, radiating from a central area. Each mouse had different platform locations that were semi-randomly determined. The platform locations remained fixed throughout the experiment. There was never a platform in the arm from which the mouse was released (constant start arm for all subjects across all trials) and no more than two adjacent arms had a platform.

The mouse was released into the start arm of the maze facing the central area. For every trial, a subject was allowed 120 sec to swim through the maze and locate a platform. If the time expired before the animal found a platform, the mouse was guided to the nearest platform. The experimenter recorded arm entries in order. Once a platform was found, the mouse remained on it for 20 sec and was then returned to a heated cage for 90 sec. The platform was removed and the mouse was subsequently released into the maze from the start arm, and allowed 120 sec to locate another platform. A session consisted of this sequence of events repeated until all four platforms were located. Each subject received one session (4 trials) per day for 12 days. Day 0 was a swim test including velocity testing and sight testing to ensure that all animals were physically capable of performing in the maze. Days 1-3 were considered training sessions since the animals had no previous experience in water or the maze. Days 4–12 were testing sessions. In order to perform well in this task mice needed to: (1) remember and avoid entering arms that had never contained platforms in the past (reference memory); (2) remember and enter the arms that should have platforms (reference memory); and (3) remember not to reenter an arm which had previously contained a platform during a particular trial/session since the platform would now be absent (working memory).

As trials increased within a session, the memory load, or the elements of information to be remembered (previously located platformed arms), increased as well. Errors were quantiﬁed for each trial within each daily testing session. Working memory errors were the number of ﬁrst and repeat entries into any arm from which a platform had been previously removed during that session [[5](#_ENREF_5)]. Reference memory errors were the number of entries into an arm where no platform had ever been. Start arm errors were scored when an animal returned to the release spot (arm 5). Errors were scored each time the subject entered the arm not containing the platform. An arm entry was defined as the mouse's whole body moving past the threshold of the entrance to an arm. Entries into the start-arm were considered reference memory errors, but were also considered separately since MRL/lpr mice performed particularly poorly in this regard. Start-arm errors are usually eliminated very quickly since there is never a platform in that arm (start-arm errors typically remain <1.0 in late trial days). Data are presented as the average errors per block, similar to the methods described in [[6](#_ENREF_6)].

**Novel object recognition**

Recognition memory tasks utilize animal tendency to spend more time exploring novel objects compared to familiar objects in order to satisfy their innate exploratory instinct. The test apparatus consisted of clean plastic rodent house measuring 25 cm × 48 cm x 22 cm, with all walls covered by black felt to reduce ambient distractions. All sessions were video-recorded. On days 1 and 2, the animal was habituated to the learning environment to reduce anxiety. The animal was allowed to explore the open cage (no objects present) for a 5 minute time period each day. On day 3 the animals were each exposed to a 3 minute familiarization session (identical objects present, A/A). This session was followed by a 90 min delay during which the animals were returned to their home cages. After the delay the animals performed a 3 minute testing session (A/B, 90 min retention trial). On day 4, the animals were again tested with a second new object (A/C, 24h retention trial). All objects were made of hard plastic and counterbalanced to control for preference bias. A mouse could not displace an object. The time to first contact, total amount of time spent with each object, on each side of the cage, as well as number of contacts were recorded and scored. The time spent was operationally defined as occurring when an animal directed its nose to the object at a distance <1.0 cm and/or by the animal touching the object with its nose or mouth. Data are presented as the *D*^2^discrimination index. The *D*^2^index is a common measure of discrimination between novel and familiar objects, and is considered one of the most reliable measures of discrimination because it corrects for total exploratory activity of each animal [[7](#_ENREF_7)]. The *D*^2^index is calculated for an A/B or A/C session by examining the difference in time spent exploring the novel and familiar objects divided by the total exploration time for both objects: D^2^ = (T_n_-T_f_ )/Total time.

1. Crusio WE, Schwegler H: **Learning spatial orientation tasks in the radial-maze and structural variation in the hippocampus in inbred mice.** *Behav Brain Funct* 2005, **1:**3.

2. Lockrow J, Boger H, Bimonte-Nelson H, Granholm AC: **Effects of long-term memantine on memory and neuropathology in Ts65Dn mice, a model for Down syndrome.** *Behav Brain Res* 2010.

3. Bimonte HA, Denenberg VH: **Estradiol facilitates performance as working memory load increases.** *Psychoneuroendocrinology* 1999, **24:**161-173.

4. Hyde LA, Hoplight BJ, Denenberg VH: **Water version of the radial-arm maze: learning in three inbred strains of mice.** *Brain Res* 1998, **785:**236-244.

5. Jarrard LE, Okaichi H, Steward O, Goldschmidt RB: **On the role of hippocampal connections in the performance of place and cue tasks: comparisons with damage to hippocampus.** *Behav Neurosci* 1984, **98:**946-954.

6. Alamed J, Wilcock DM, Diamond DM, Gordon MN, Morgan D: **Two-day radial-arm water maze learning and memory task; robust resolution of amyloid-related memory deficits in transgenic mice.** *Nat Protoc* 2006, **1:**1671-1679.

7. Sik A, van Nieuwehuyzen P, Prickaerts J, Blokland A: **Performance of different mouse strains in an object recognition task.** *Behav Brain Res* 2003, **147:**49-54.
