# Supplementary material for: Estrogen receptor alpha deficiency protects against development of cognitive impairment in murine lupus
Source: J Neuroinflammation. 2014 Dec 16;11:171. doi: 10.1186/s12974-014-0171-x (PMC4272530; doi:10.1186/s12974-014-0171-x)
Supplement: Supplementary file 2 — (1) Summary of reference memory errors: 86% of MRL/lpr ERKO mice improved (vs. 42% of WT). (2) Summary of working memory incorrect errors: 74% of MRL/lpr ERKO mice improved (vs. 48% of WT). (3) Summary of working memory correct errors: 62% of MRL/lpr ERKO mice improved (vs. 46% of WT). Figure S1. Western blot analysis of whole cell lysates from hippocampus and cortex of MRL/lpr and MRL/lpr ERαKO mice for Zo-1. There were no significant global changes in Zo-1 at 10 weeks of age in the hippocampus or cortex of MRL/lpr vs. MRL/lpr ERαKO mice although there was a trend towards reduced Zo-1 in MRL/lpr ERαKO mice. Figure S2. Western blot analysis of whole cell lysates from hippocampus of MRL/lpr and MRL/lpr ERαKO mice (n=9 WT, 9 KO) for (A) GFAP (marker of astrocytosis), (B) Iba1 (marker of microgliosis), and (C) MAP2 (marker of neuronal growth, plasticity, degeneration). There were no significant global changes in these markers at 10 weeks of age in the hippocampus of MRL/lpr vs. MRL/lpr ERαKO mice. Figure S3. Hippocampal MAP2 staining in MRL/lpr and MRL/lpr ERαKO mice. IHC images demonstrate multiple MRL/lpr animals had reduced overall MAP-2 signal in the hippocampus, suggesting reduced numbers of dendrites and dendritic complexity in MRL/lpr versus MRL/lpr ERαKO mice. Scale bar in lower right corner represents 50 microns. Figure S4. Hippocampal Iba1 staining in MRL/lpr and MRL/lpr ERαKO mice. IHC images demonstrate diffuse glial activation and clustered microglia in the dentate of both MRL/lpr and MRL/lpr ERαKO mice. These 20× images demonstrate increased Iba1 immunopositive cells in MRL/lpr WT versus ERαKO mice. Scale bar in lower right corner represents 50 microns. [file 12974_2014_171_MOESM2_ESM.docx]

**Supplemental Data**

Table 1 – Mean number of Reference Memory errors ± SE


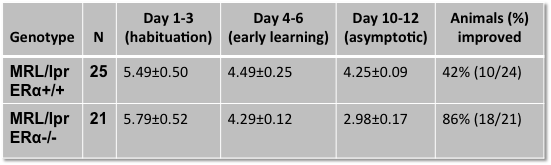

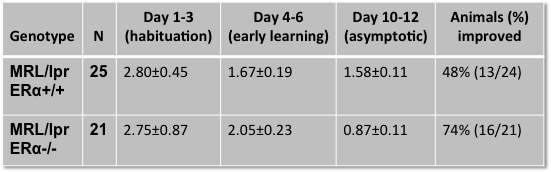


Table 2 – Mean number of Working Memory Incorrect errors ± SE

Table 3 – Mean number of Working Memory Correct errors ± SE


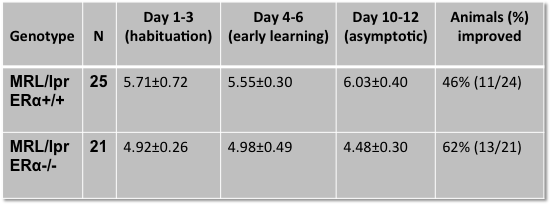


**S1**


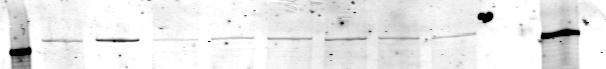

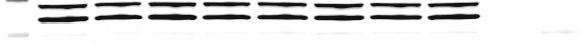

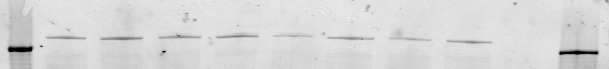

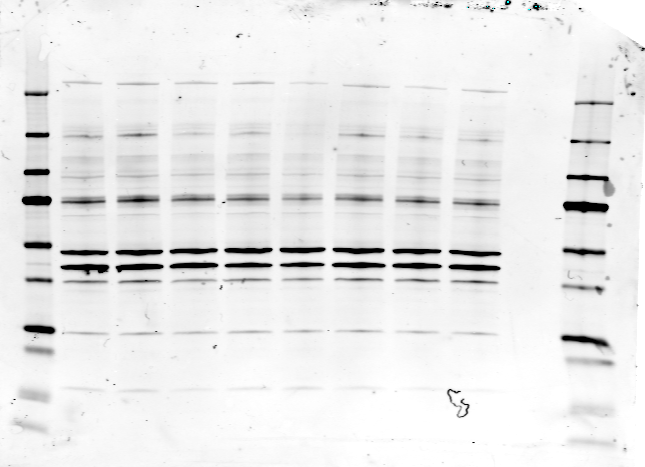


Zo-1

(225kDa)

actin


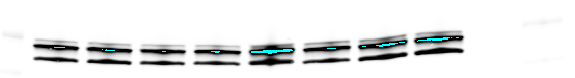


GFAP

actin

50 kDa

46 kDa


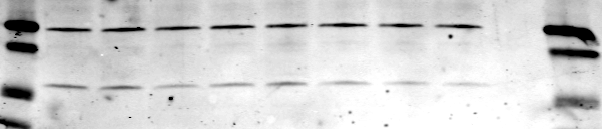

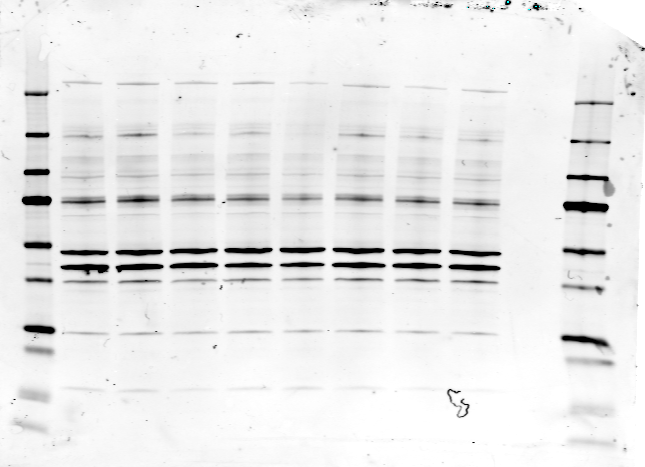


46 kDa

actin

17 kDa

Iba1

**S2A**

**S2B**

MAP2

actin


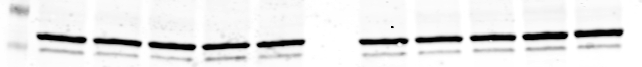

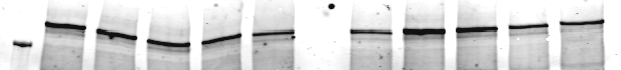


250 kDa

46 kDa

**S2C**

**S3**

MAP2 in hippocampus

**ERαKO**

**ERαWT**


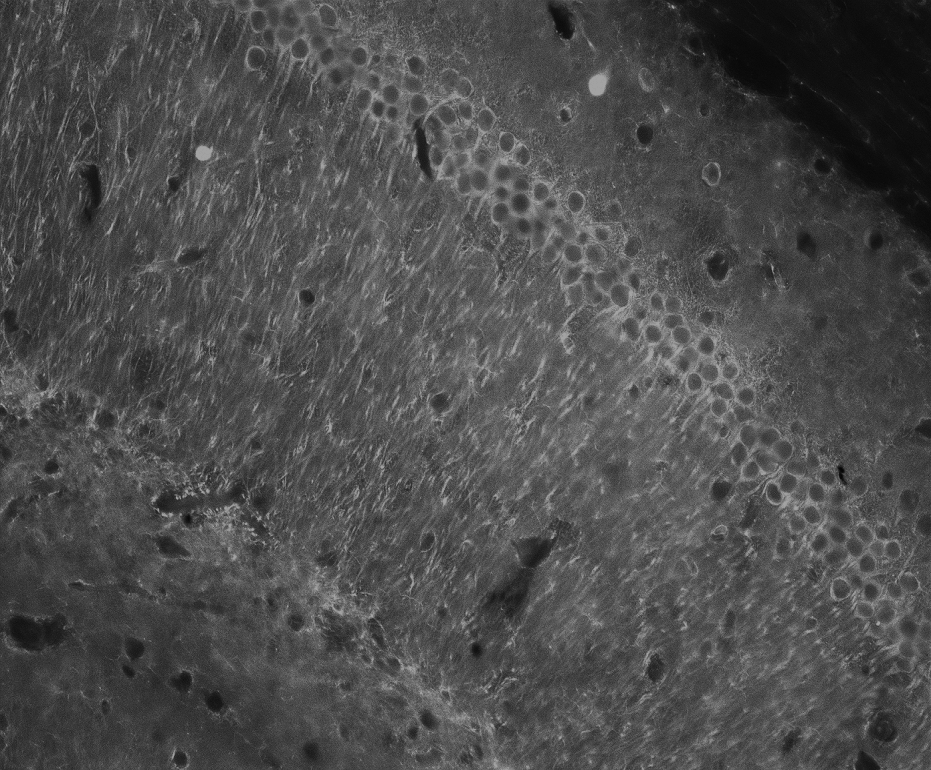

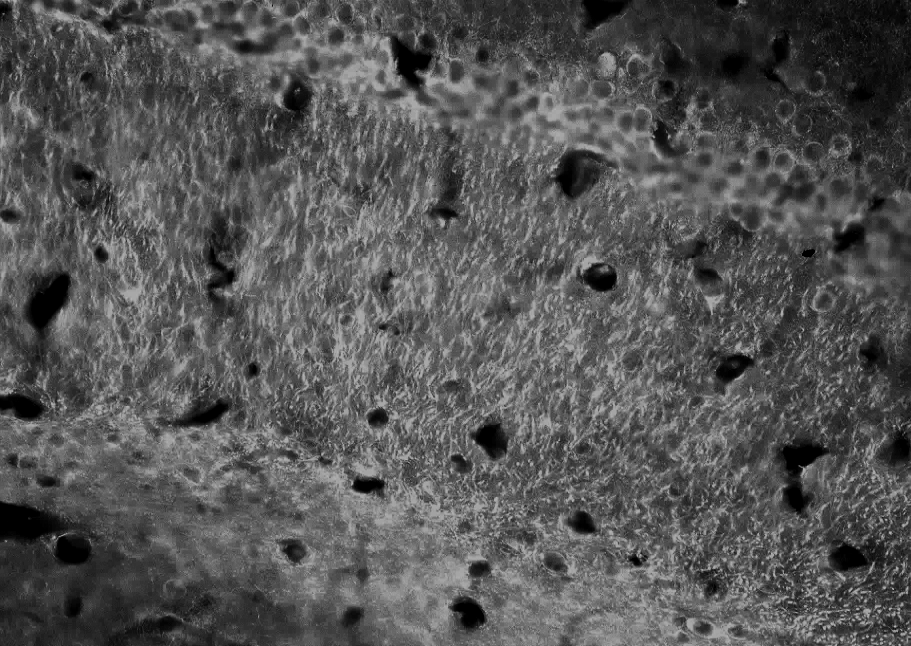


Scale bar in lower right corner represents 100 microns


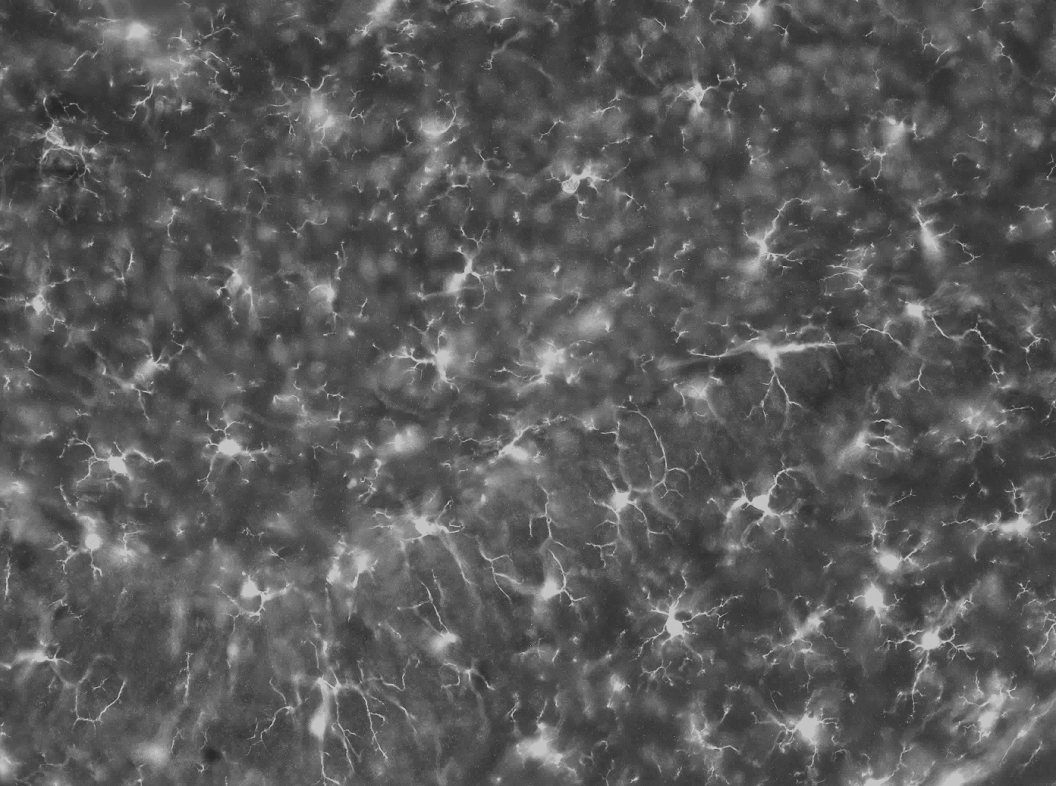

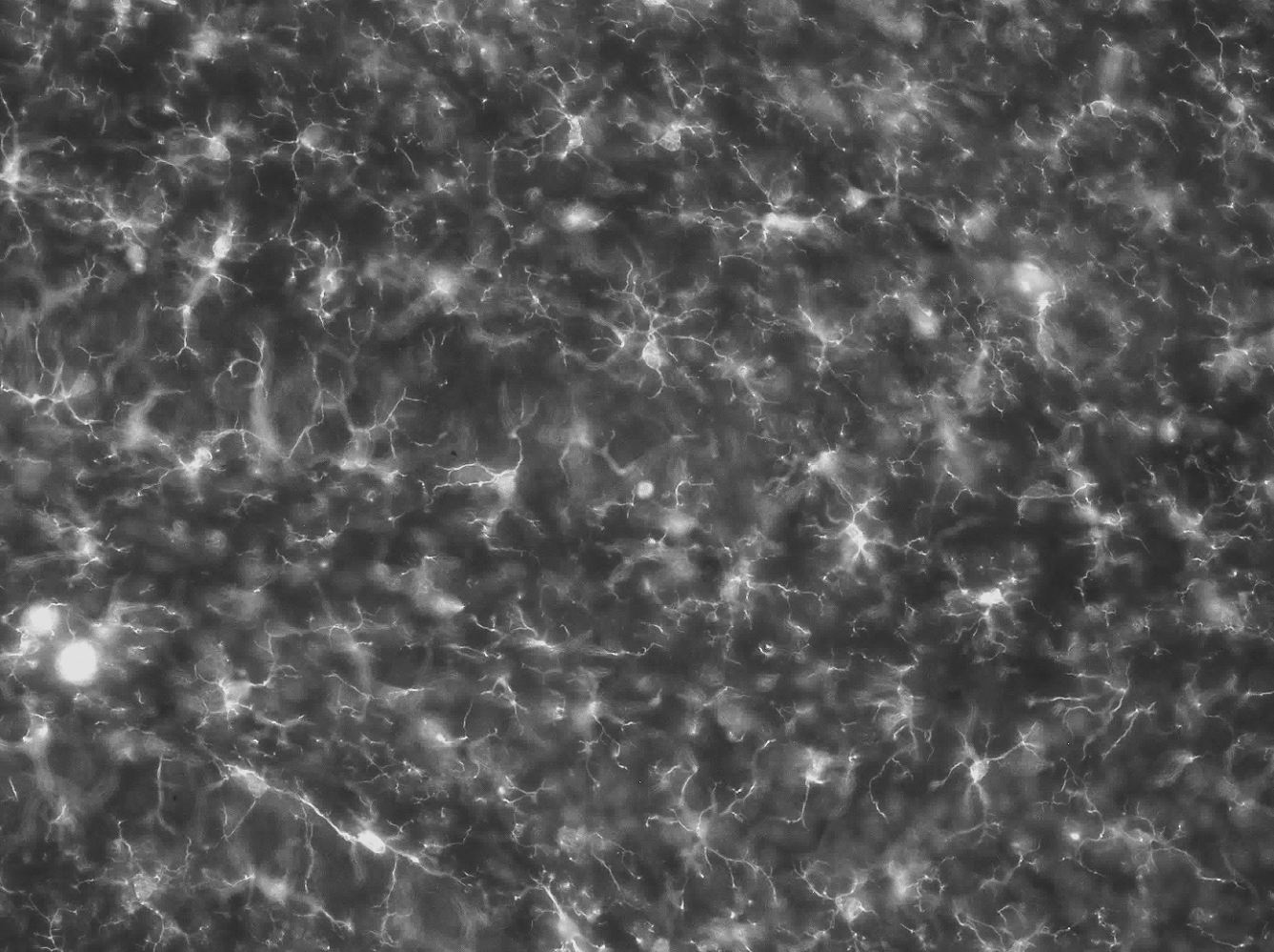

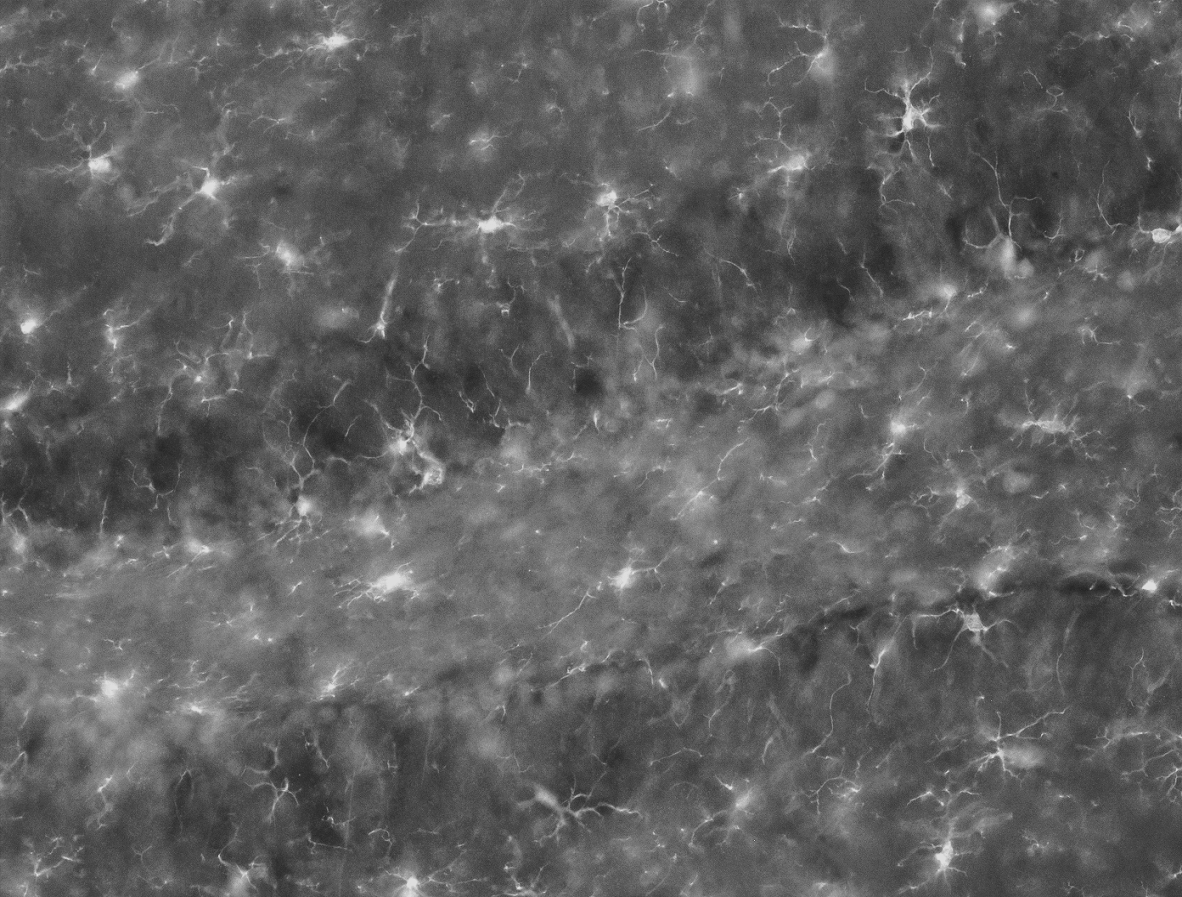

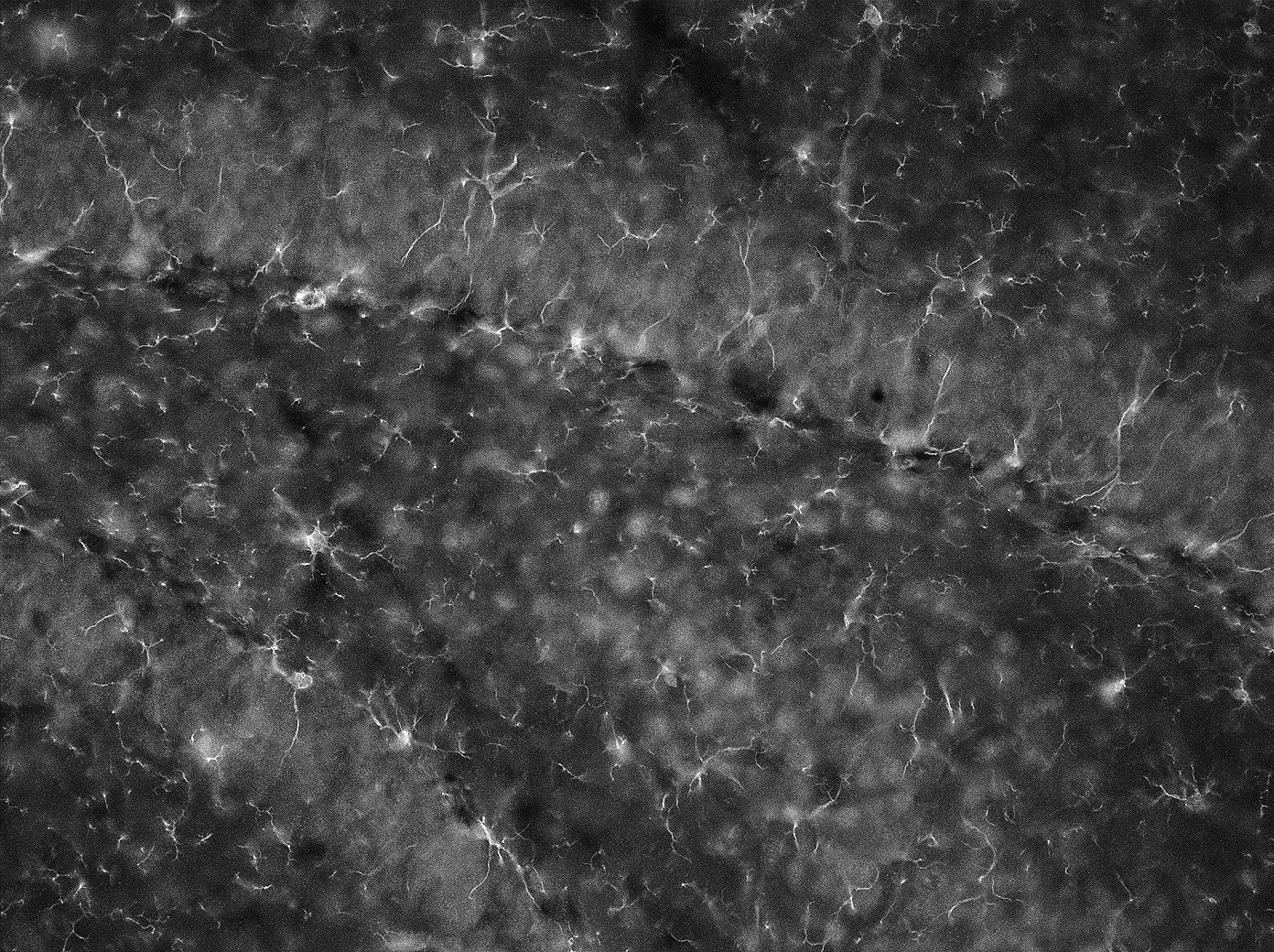

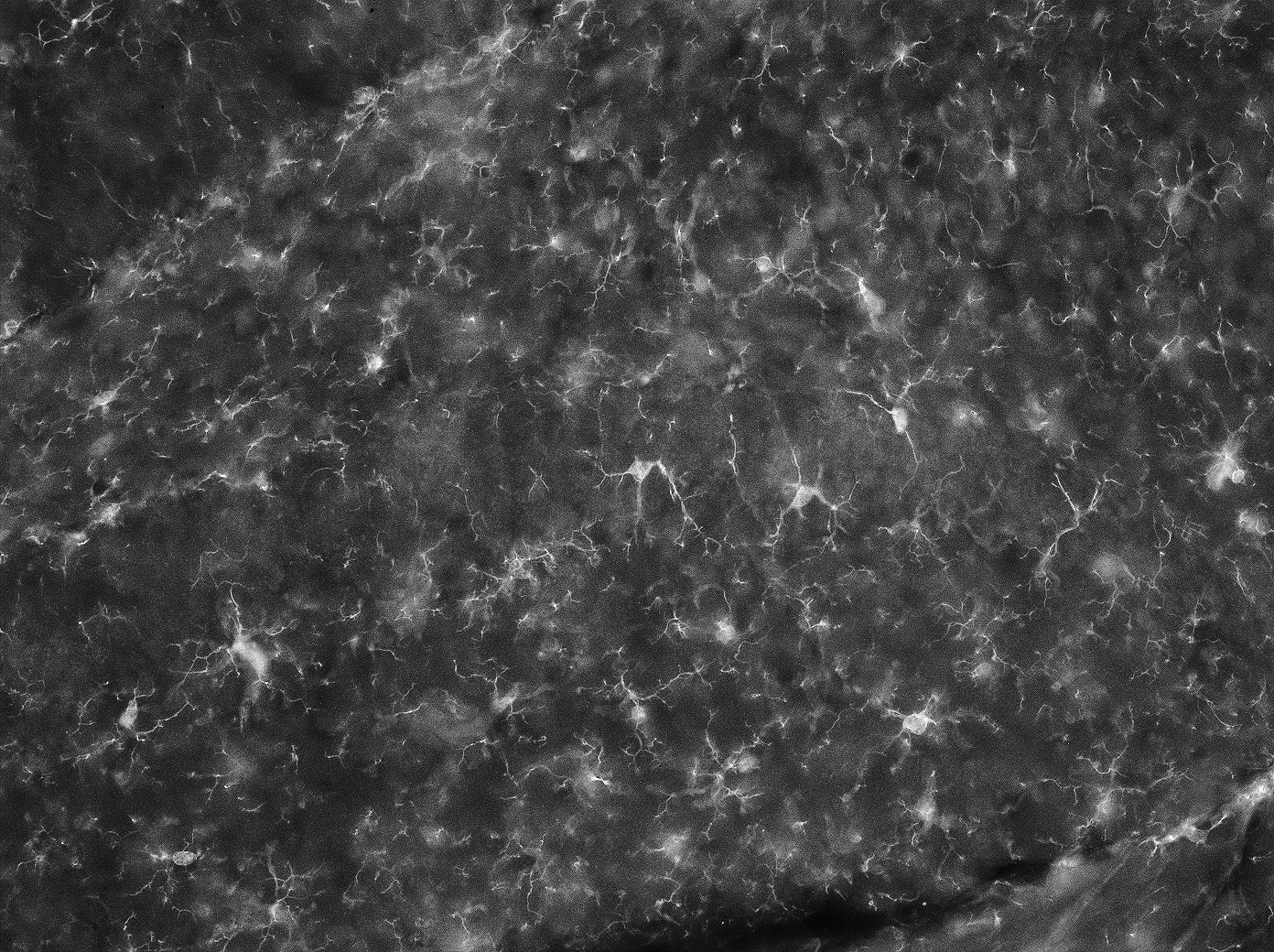

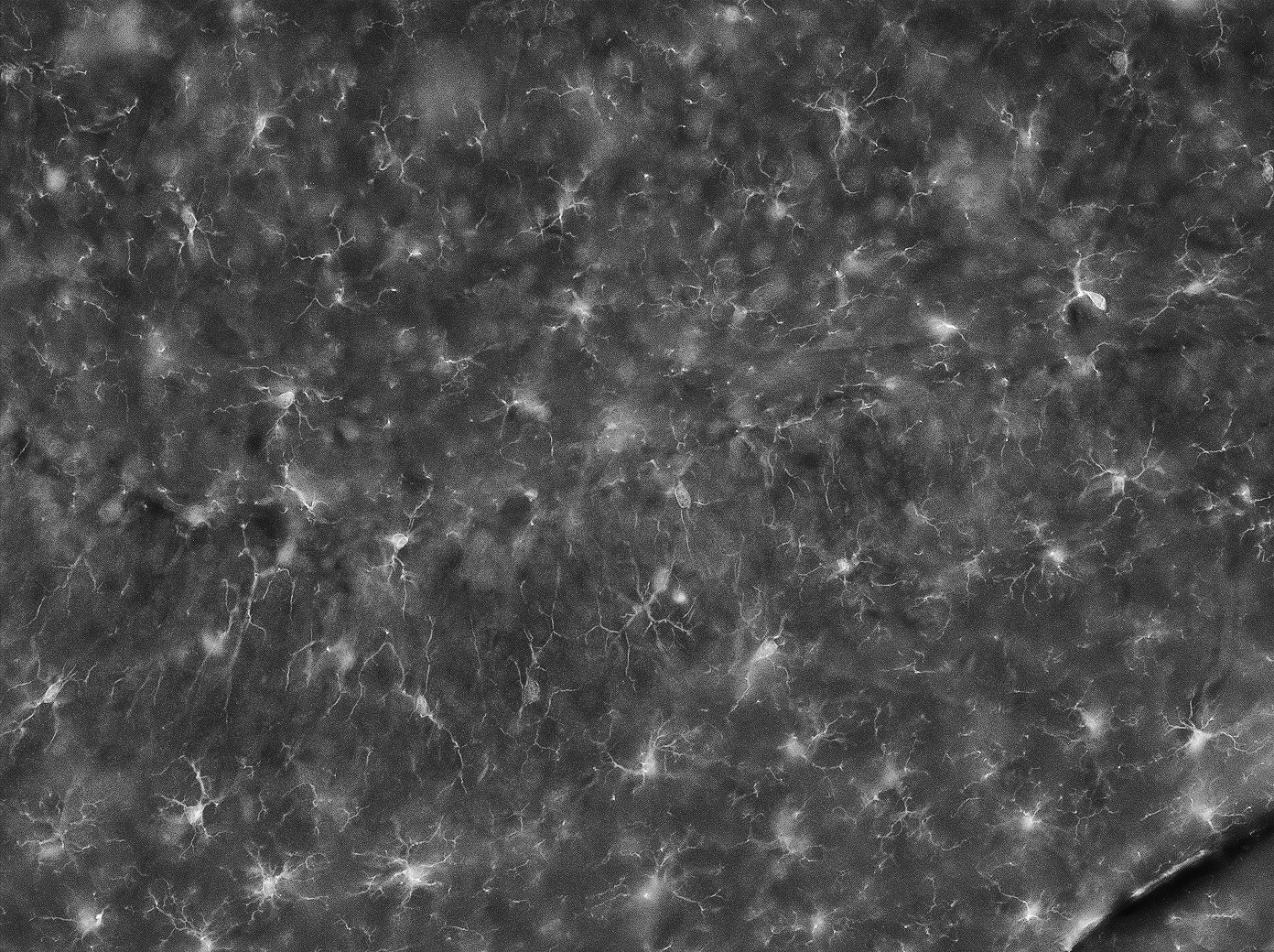


**ERαWT**

**ERαKO**

**S4**

Iba1 – Dentate 20x

Scale bar in lower right corner represents 50 microns
